# A pan-cancer analysis reveals the diagnostic and prognostic role of CDCA2 in low-grade glioma

**DOI:** 10.1371/journal.pone.0291024

**Published:** 2023-09-21

**Authors:** Wenle Li, Dong Lv, Jieqin Yao, Boxian Chen, Huanqiang Liu, Wensheng Li, Chengjie Xu, Zhenzhe Li

**Affiliations:** 1 Department of Gynecology, Affiliated Hospital of Guangdong Medical University, Zhanjiang, Guangdong, China; 2 Department of Psychiatry, Affiliated Hospital of Guangdong Medical University, Zhanjiang, Guangdong, China; 3 Department of Neurosurgery, Affiliated Hospital of Guangdong Medical University, Zhanjiang, Guangdong, China; 4 Department of Neurosurgery, Central People’s Hospital of Zhanjiang, Zhanjiang, Guangdong, China; University of Illinois at Urbana-Champaign, UNITED STATES

## Abstract

**Background:**

Cell division cycle associated 2 (CDCA2), a member of the cell division cycle associated proteins (CDCA) family, is crucial in the regulation of cell mitosis and DNA repair. CDCA2 was extensively examined in our work to determine its role in a wide range of cancers.

**Methods:**

CDCA2 differential expression was studied in pan-cancer and in diverse molecular and immunological subgroups in this research. Additionally, the diagnostic and prognostic significance of CDCA2 in pan-cancer was also evaluated using receiver operating characteristic (ROC) and Kaplan–Meier (KM) curves. Prognostic value of CDCA2 in distinct clinical subgroups of lower grade glioma (LGG) was also investigated and a nomogram was constructed. Lastly, potential mechanisms of action of CDCA2 were interrogated including biological functions, ceRNA networks, m6A modification and immune infiltration.

**Results:**

CDCA2 is shown to be differentially expressed in a wide variety of cancers. Tumors are diagnosed and forecasted with a high degree of accuracy by CDCA2, and the quantity of expression CDCA2 is linked to the prognosis of many cancers. Additionally, the expression level of CDCA2 in various subgroups of LGG is also closely related to prognosis. The results of enrichment analyses reveal that CDCA2 is predominantly enriched in the cell cycle, mitosis, and DNA replication. Subsequently, hsa-miR-105-5p is predicted to target CDCA2. In addition, 4 lncRNAs were identified that may inhibit the hsa-miR-105-5p/CDCA2 axis in LGG. Meanwhile, CDCA2 expression is shown to be associated to m6A-related genes and levels of immune cell infiltration in LGG.

**Conclusion:**

CDCA2 can serve as a novel biomarker for the diagnosis and prognosis in pan-cancer, especially in LGG. For the development of novel targeted therapies in LGG, it may be a potential molecular target. However, to be sure, we’ll need to do additional biological experiments to back up our results from bioinformatic predictions.

## Introduction

CDCA2, also known as Repo-Man (recruits PP1 onto mitotic chromatin at anaphase), belongs to the CDCA protein family [1]. Several studies have found that CDCA2 plays an important role in the regulation of DNA damage response in the cell cycle by binding to protein phosphatase 1γ (PP1γ) [2]. It has been shown that Repo-Man is a modular protein regulated/targeted by PP1γ that coordinates chromatin remodeling and nuclear membrane reorganization during anaphase of mitosis. Chromatin is remodeled by the C-terminal Repo-Man module, which directs PP1 to amitotic chromosomes and controls histone H3 dephosphorylation [3]. Additionally, CDCA2 facilitates the dephosphorylation of histone H3 during mitosis in a way that is reliant on PP1 [4, 5].

Several recent investigations have indicated that abnormal expression of CDCA2 contributes to the occurrence and development of some malignancies. For example, in oral squamous cell carcinoma, CDCA2 is often overexpressed. It keeps the cell cycle from stopping in the G1 phase by lowering CDKI expression and controlling the DNA damage response [6]. Furthermore, studies have shown that CDCA2 is increased in several malignancies, including neuroblastoma [7], melanoma, and kidney cancer [8, 9].

Glioma is the most prevalent malignant brain tumor [10]. The World Health Organization (WHO) has established four distinct grades for the classification of gliomas. Additionally, the Cancer Genome Atlas (TCGA) has categorized grade II and III gliomas as lower-grade glioma (LGG). While LGG exhibits a higher survival rate compared to glioblastoma (GBM), certain patients may experience progression to GBM within a few months. Conventional treatments for glioma, including surgery, chemotherapy, and radiotherapy, have demonstrated limited efficacy [11]. Therefore, it is essential to investigate a new predictive biomarker in order to advance targeted therapy for low-grade gliomas.

However, to the best of our knowledge, no systematic and comprehensive studies on the diagnosis, prognosis, and related biological functions of CDCA2 in pan-cancer, especially in LGG have been reported. Our study indicates that CDCA2 may not only serve as a promising biomarker for the diagnosis and prognosis of diverse cancers, but also as an appealing target for LGG therapy.

## Materials and methods

### CDCA2 expression analysis

Download RNA-seq data for 33 tumor types and corresponding normal tissues from The Cancer Genome Atlas (TCGA) database and the Genotype Tissue Expression (GTEx) database. R software v3.6.3 was used to determine CDCA2 gene expression differences between unpaired normal and cancerous tissues, as well as the expression differences in paired tumor and normal tissue samples, and p < 0.05 was regarded as statistically significant. Log2 (value+1) was used to normalize the data. The Wilcoxon rank sum test was used for expression analysis. Besides, the TISIDB (http://cis.hku.hk/TISIDB) [12] was used to study the associations between CDCA2 expression and molecular or immunological subtypes in pan-cancer (ns, p ≥ 0.05; *, p < 0.05; **, p < 0.01; ***, p < 0.001).

### Diagnostic value analysis

ROC curves as well as the area under the ROC curves (AUC) were used to assess the diagnostic performance of CDCA2. Tumor data from TCGA and normal tissue data from GTEx were used for ROC analysis. AUC greater than 0.9 indicated very good performance, greater than 0.8 indicated good performance, and greater than 0.7 indicated useful discrimination.

### Survival prognosis analysis

The KM curves were used in the survival analysis to examine the relationship between CDCA2 expression and OS (overall survival), DSS (disease specific survival) and PFI (progress free interval), respectively. In addition, the prognosis of the different clinical subgroups of LGG was further analyzed. The criteria for “high” and “low” expressions for the KM analysis were based on the tumor cohort median. Survival analysis and visualization were carried out using the R packages “survival (v3.2.1)” and “survminer (v3.3.3)”, and p < 0.05 is considered statistically significant.

### Construction and validation of the nomogram

Univariate and multivariate Cox regression analyses were performed to identify potential prognostic variables for OS in patients with LGG. Based on these independent prognostic factors, a prognostic nomogram was established for predicting the probability of 1-, 2-, and 3-year OS for LGG patients. Then, calibration plots were used to assess the nomogram’s performance. All analyses were conducted via the “survival (v3.2.1)”, “ggplot2 (v3.3.3)”, and “rms (v6.2.0)” R packages, and p < 0.05 was deemed statistically significant.

### Enrichment analyses

In the present study, Co-expression analysis of CDCA2 was performed by the R package “stat”. The criteria for the positive and negative co-expressed genes were based on Spearman correlation. |Cor|>0.3 and p value<0.05 were settled for cutoffs. Gene Ontology (GO) and Kyoto Encyclopedia of Genes and Genomes (KEGG) enrichment analyses of the top 100 positive and negative co-expressed genes were conducted using the R packages “ggplot2 (v3.3.3)” and “clusterProfiler (v4.4.4)”. Single gene differential analysis of CDCA2 was performed, and gene set enrichment analysis (GSEA) of these differentially expressed genes was performed using the R package “clusterProfiler (v4.4.4). The NES top 10 gene sets satisfying the threshold (p.adj<0.05 and qvalue<0.25) were selected for visualization. The R packages “DESeq2 (v1.36.0)” and “clusterProfiler (v4.4.4)” were used for statistics and visualization.

### Construction of the ceRNA network

The upstream miRNAs potentially binding to CDCA2 were predicted by starbase (https://starbase.sysu.edu.cn/index.php). The setting parameters are: Clade (mammal), Genome (human), Assembly (hg19), miRNA (all), CLIP-Data (≥1), Degradome-Data (with or without data), pan-Cancer (1 cancer type), program Num (1 program), target (CDCA2). Further investigation was limited to miRNAs that showed up in more than one prediction program.

In addition, potential lncRNAs bound to miRNA-105-5p were predicted using miRNet (https://www.mirnet.ca/). Then, these lncRNAs were intersected with lncRNAs retrieved from the starBase database to obtain the lncRNAs with the most potential to regulate miRNA-105-5p.

Subsequently, a ceRNA network was constructed by the R package “ggalluvial”.

### m6A modification in LGG

The relationships between CDCA2 expression and m6A regulators (YTHDF1, YTHDF2, YTHDF3, YTHDC1, YTHDC2, IGF2BP1, IGF2BP2, IGF2BP3, HNRNPA2B1, HNRNPC, RBMX, ZC3H13, METTL14, METTL3, RBM15, RBM15B, VIRMA, WTAP, FTO, and ALKBH5) [13] expression in LGG were investigated by the Spearman correlation coefficient. Differential expression of m6A regulators in the high and low CDCA2 expression groups was analyzed using the Wilcoxon rank sum test. The criteria for “high” and “low” CDCA2 expression groups were based on the CDCA2 expression median.

### Immune infiltration

Immune cell infiltration levels and correlations between immune score, stroma score, estimate score, and CDCA2 expression were determined. Spearman correlation was used for revelance screening. And 0<|Cor|<0.3 represents weak or no correlation; 0.3<|Cor|<0.5 represents weak correlation; 0.5<|Cor|<0.8 represents moderate correlation; 0.8<|Cor|<1 represents strong correlation. Besides, differences in immune cell enrichment scores between the CDCA2 high and low expression groups were explored in LGG. These analyses were performed using the R packages “GSVA” and “estimate”, and p<0.05 was considered to be statistically significant. Furthermore, to explore the relationship between immune cell infiltration levels and the prognosis of LGG patients, survival analyses were performed by the immune module of TIMER (http://timer.cistrome.org/).

## Results

### CDCA2 expression in pan-cancer

**Fig 1:**
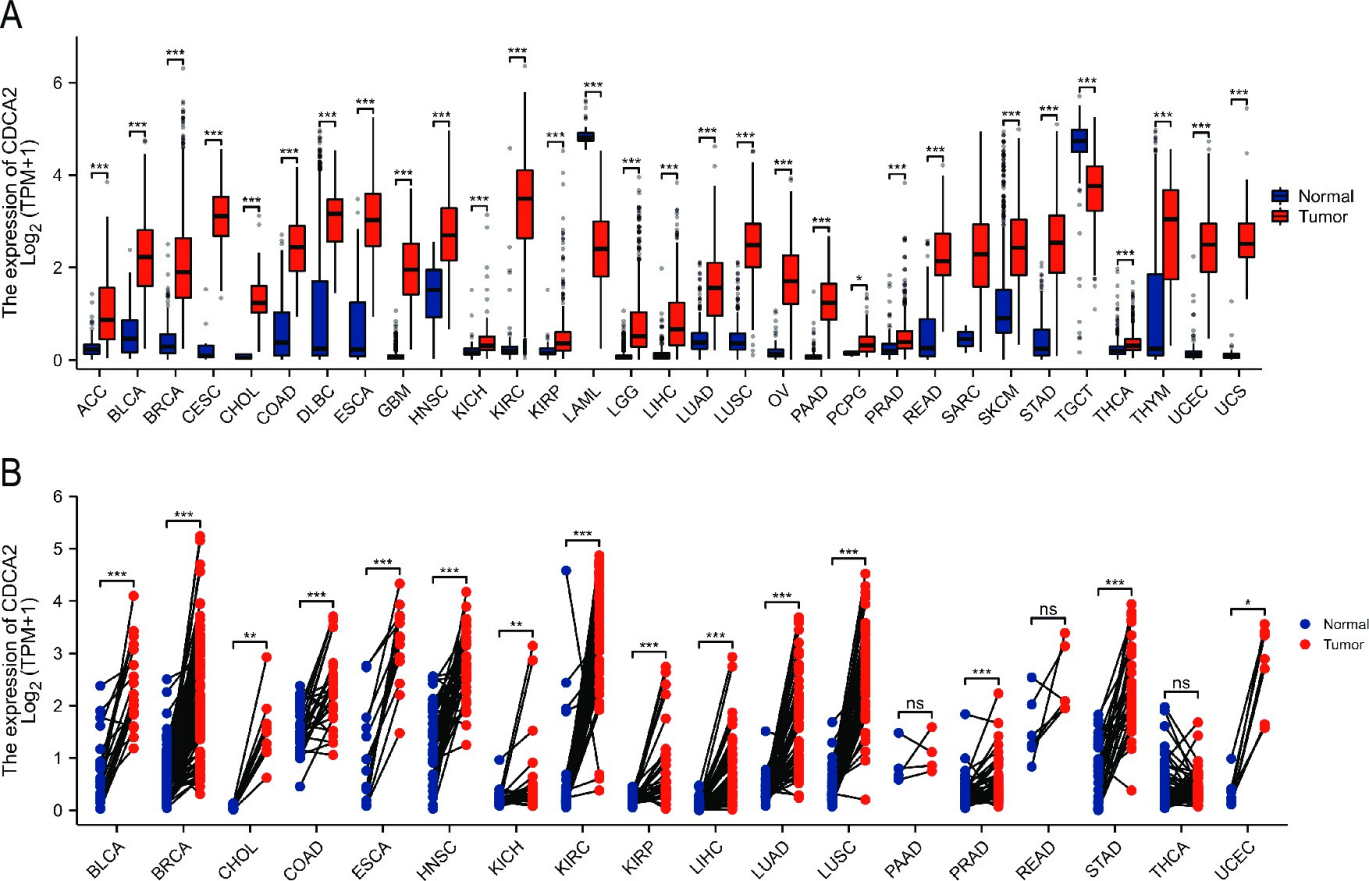
Expression level of CDCA2 in normal and tumor tissues. (A) CDCA2 expression in TCGA tumors and GTEx normal tissues; (B) The expression of CDCA2 in tumors and adjacent normal tissues in TCGA (*p < 0.05, **p < 0.01, ***p < 0.001).

Differences in CDCA2 expression between tumors (from the TCGA database) and normal tissues (from GTEx database) were compared. CDCA2 expression levels were shown to be noticeably upregulated in adrenocortical carcinoma (ACC), bladder urothelial carcinoma (BLCA), breast invasive carcinoma (BRCA), cervical squamous cell carcinoma and endocervical adenocarcinoma (CESC), cholangiocarcinoma (CHOL), colon adenocarcinoma (COAD), diffuse large B-cell lymphoma (DLBC), esophageal carcinoma (ESCA), glioblastoma (GBM), head and neck squamous cell carcinoma (HNSC), kidney chromophobe (KICH), kidney renal clear cell carcinoma (KIRC), kidney renal papillary cell carcinoma (KIRP), lower grade glioma (LGG), liver hepatocellular carcinoma (LIHC), lung adenocarcinoma (LUAD), lung squamous cell carcinoma (LUSC), ovarian serous cystadenocarcinoma (OV), pancreatic adenocarcinoma (PAAD), pheochromocytoma and paraganglioma (PCPG), prostate adenocarcinoma (PRAD), rectum adenocarcinoma (READ), skin cutaneous melanoma (SKCM), stomach adenocarcinoma (STAD), thyroid carcinoma (THCA), thymoma (THYM), uterine corpus endometrial carcinoma (UCEC), uterine carcinosarcoma (UCS), while downregulated in acute myeloid leukemia (LAML), testicular germ cell tumors (TGCT) (Fig 1A). Gene expression levels are different between different individuals, and paired samples can better reflect the specific molecular expression differences in the same individual. Thus, the expression of CDCA2 in paired normal and tumor tissues from the TCGA database was also analyzed. The results revealed that CDCA2 expression was significantly higher in BLCA, BRCA, CHOL, COAD, ESCA, HNSC, KICH, KIRC, KIRP, LIHC, LUAD, LUSC, PRAD, STAD, and UCEC (Fig 1B).

### CDCA2 expression in immune and molecular subtypes of cancers

**Fig 2:**
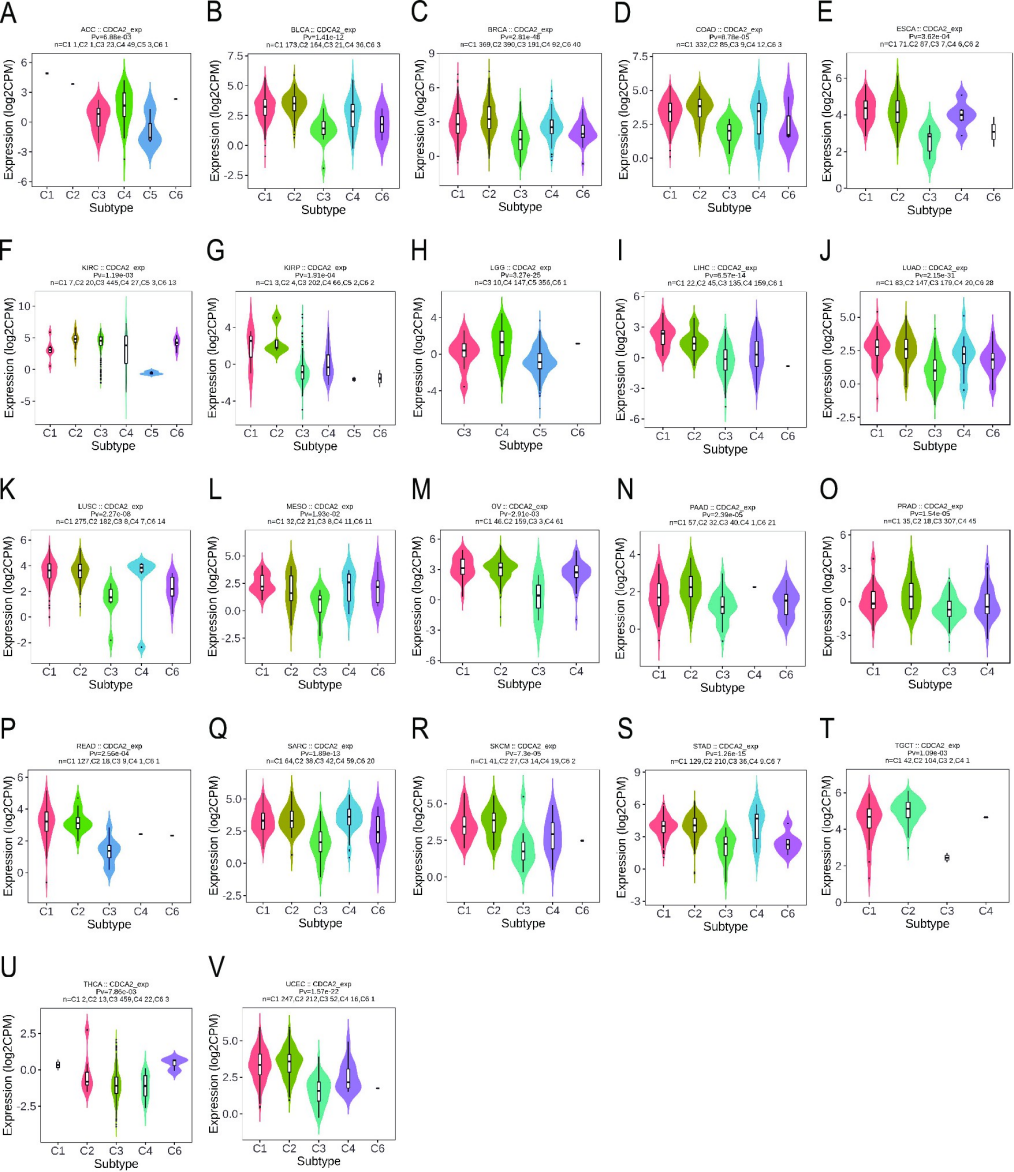
Correlations between CDCA2 expression and immune subtypes across TCGA tumors. (A) ACC; (B) BLCA; (C) BRCA; (D) COAD; (E) ESCA; (F) KIRC; (G) KIRP; (H) LGG; (I) LIHC; (J) LUAD; (K) LUSC; (L) MESO; (M) OV; (N) PAAD; (O) PRAD; (P) READ; (Q) SARC; (R) SKCM; (S) STAD; (T) TGCT; (U) THCA; (V) UCEC.

The TISIDB website was used to investigate the differential expression of CDCA2 in distinct molecular and immunological subtypes of pan-cancer. Thorsson et al. have introduced a novel worldwide immune classification system for solid tumors [14]. Six distinct immune subtypes (ISs) were identified. The wound healing (C1) showed an elevated expression of angiogenic genes, a high proliferation rate and a low Th1/Th2 ratio related to the adaptive immune infiltrate. The IFN-g dominant (C2) presented a high proliferation rate, the highest intratumoral heterogeneity, macrophages M1/M2 polarisation and CD8 T cell population and the greatest T-cell receptor (TCR) diversity. The inflammatory (C3) was defined by elevated Th17 and Th1 genes, low to moderate proliferation, lower levels of aneuploidy, higher somatic copy-number alterations and the most favourable prognosis. The lymphocyte depleted (C4) presented moderate cell proliferation and intratumoral heterogeneity, and a prominent macrophage signature with Th1 suppressed and a high M2 response; consistent with these features, it was associated with a poor outcome. The immunologically quiet (C5) displayed the lowest lymphocyte and highest macrophage responses, dominated by M2, and had low rates of proliferation and heterogeneity. Finally, the TGF-b dominant (C6) was a small group of mixed tumours with the highest TGF-b signature and a high lymphocytic infiltrate with a balanced Th1:Th2 ratio. Together with C4, C6 was associated with the worst prognosis. We discovered that CDCA2 expression differed among distinct immune subtypes of ACC, BLCA, BRCA, COAD, ESCA, GBM, KIRC, KIRP, LGG, LIHC, LUAD, LUSC, MESO, OV, PAAD, PRAD, READ, SARC, SKCM, STAD, TGCT, THCA, and UCEC (Fig 2).

**Fig 3:**
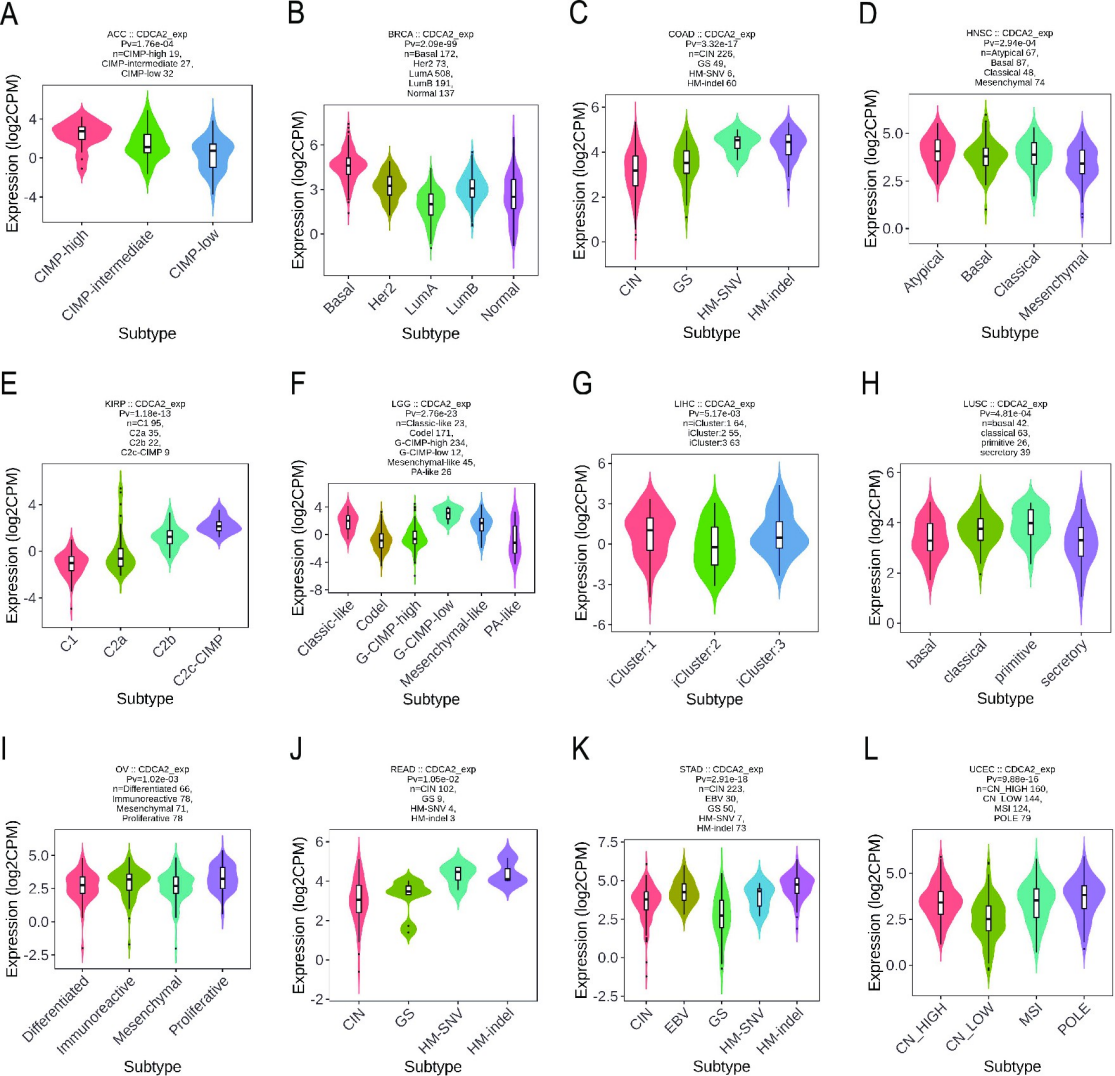
Correlations between CDCA2 expression and molecular subtypes across TCGA tumors. (A) ACC; (B) BRCA; (C) COAD; (D) HNSC; (E) KIRP; (F) LGG; (G) LIHC; (H) LUSC; (I) OV; (J) READ; (K) STAD; (L) UCEC.

Simultaneously, it was discovered that CDCA2 expression differs amongst cancer molecular subgroups, including ACC, BRCA, COAD, HNSC, KIRP, LGG, LIHC, LUSC, OV, READ, STAD, and UCEC (Fig 3). Further, for ACC, CDCA2 was identified to express the highest in the molecular subtype of CIMP-high (Fig 3A). For BRCA, CDCA2 was identified to express the highest in the molecular subtype of Basal (Fig 3B). For COAD and READ, CDCA2 was identified to express the highest in the molecular subtype of HM-SNV (Fig 3C and 3J). For HNSC, CDCA2 was identified to express the highest in the molecular subtype of Atypical (Fig 3D). For KIRP, CDCA2 expression was identified to be the highest in the C2c-CIMP molecular subtype (Fig 3E). For LGG, CDCA2 was expressed the highest in the molecular subtype of G-CIMP-low (Fig 3F). For LIHC, CDCA2 was the most highly expressed in the molecular subtype of iCluster:1 (Fig 3G). For LUSC, CDCA2 showed the highest expression in the molecular subtype of primitive (Fig 3H). For OV, CDCA2 expression was identified to be the highest in the molecular subtype of Proliferative (Fig 3I). For STAD, CDCA2 was expressed the highest in the molecular subtype of HM-indel (Fig 3K). For UCEC, CDCA2 showed the highest expression in the molecular subtype of POLE (Fig 3L).

### Diagnostic value of CDCA2 in pan-cancer

**Fig 4:**
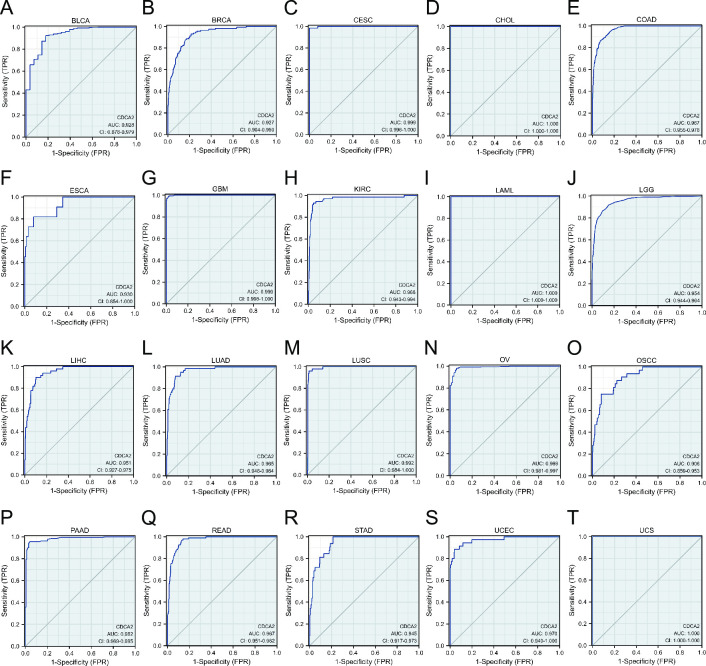
The ROC curve analysis of CDCA2 in pan-cancer (AUC > 0.9). (A) BLCA; (B) BRCA; (C) CESC; (D) CHOL; (E) COAD; (F) ESCA; (G) GBM; (H) KIRC; (I) LAML; (J) LGG; (K) LIHC; (L) LUAD; (M) LUSC; (N) OV; (O) OSCC; (P) PAAD; (Q) READ; (R) STAD; (S) UCEC; (T) UCS.

The ROC curve was utilized to assess CDCA2 diagnostic values in pan-cancer. The results showed that CDCA2 had a high predictive performance (AUC > 0.9) for 20 cancer types, including BLCA, BRCA, CESC, CHOL, COAD, ESCA, GBM, KIRC, LAML, LGG, LIHC, LUAD, LUSC, OV, OSCC, PAAD, READ, STAD, UCEC, and UCS (Fig 4). Moreover, CDCA2 also demonstrated good diagnostic prediction ability (0.9>AUC>0.7) in nine other malignancies, including ACC, DLBC, HNSC, KIRP, PRAD, SKCM, TGCT, THCA, and THYM (S1 Fig).

### Prognostic analysis of CDCA2 in cancers

**Fig 5:**
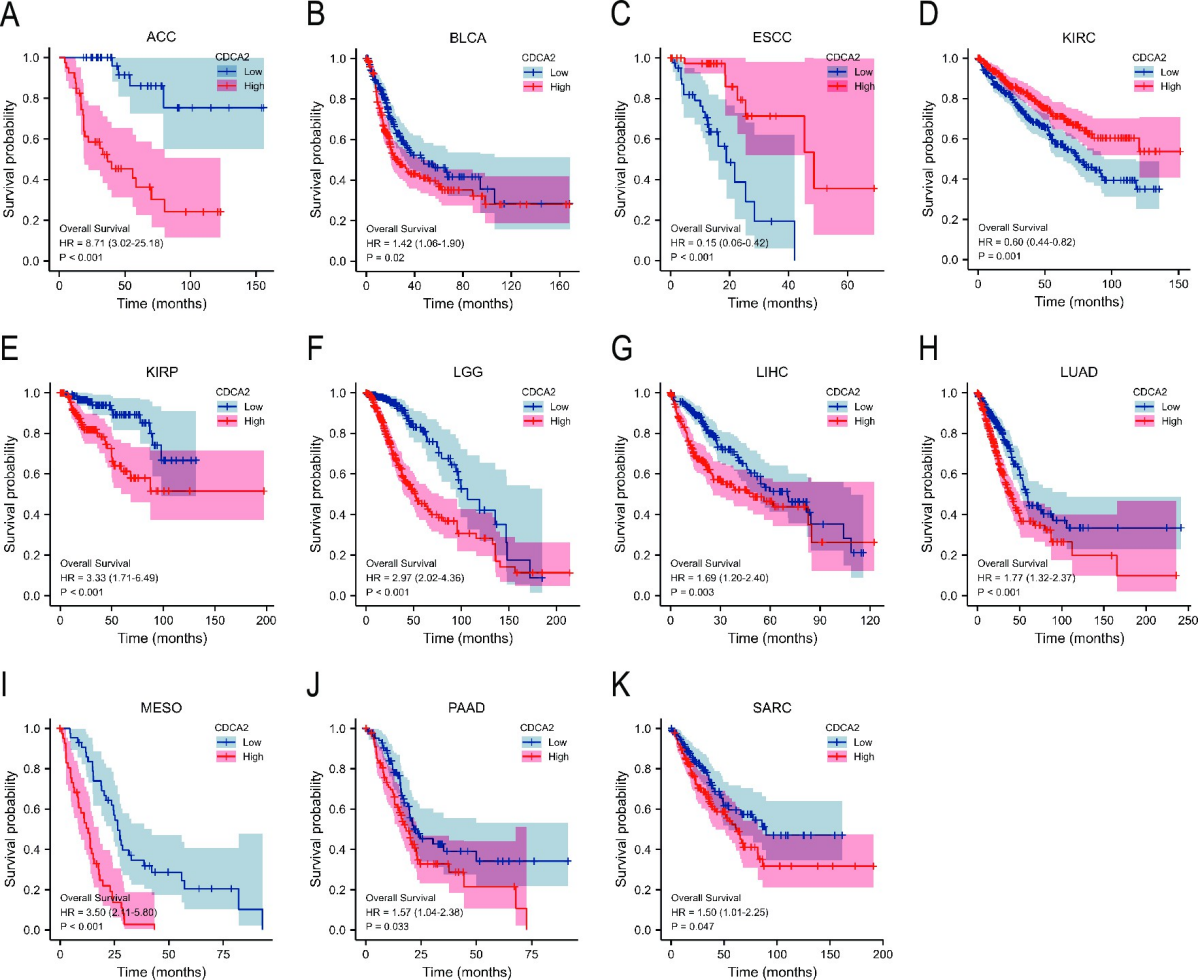
Overall survival curves of CDCA2 in cancers. (A) ACC; (B) BLCA; (C) ESCC; (D) KIRC; (E) KIRP; (F) LGG; (G) LIHC; (H) LUAD; (I) MESO; (J) PAAD; (K) SARC.

Survival analysis found that elevated CDCA2 expression was related to a poor prognosis (OS, DSS, PFI) in ACC, BLCA, ESCC, KIRC, KIRP, LGG, LIHC, LUAD, MESO, PAAD, and SARC (Fig 5, S2 and S3 Figs). Besides, the associations of CDCA2 with OS in different clinical subgroups of LGG were investigated. CDCA2 upregulation was observed to be associated with shorter overall survival in a subgroup stratified analysis of IDH status (Mut), WHO grade (G3), histological type (astrocytoma and oligodendroglioma), and primary therapy outcome (PD and SD) (S4 Fig).

### Univariate and multivariate analyses in LGG

**Fig 6:**
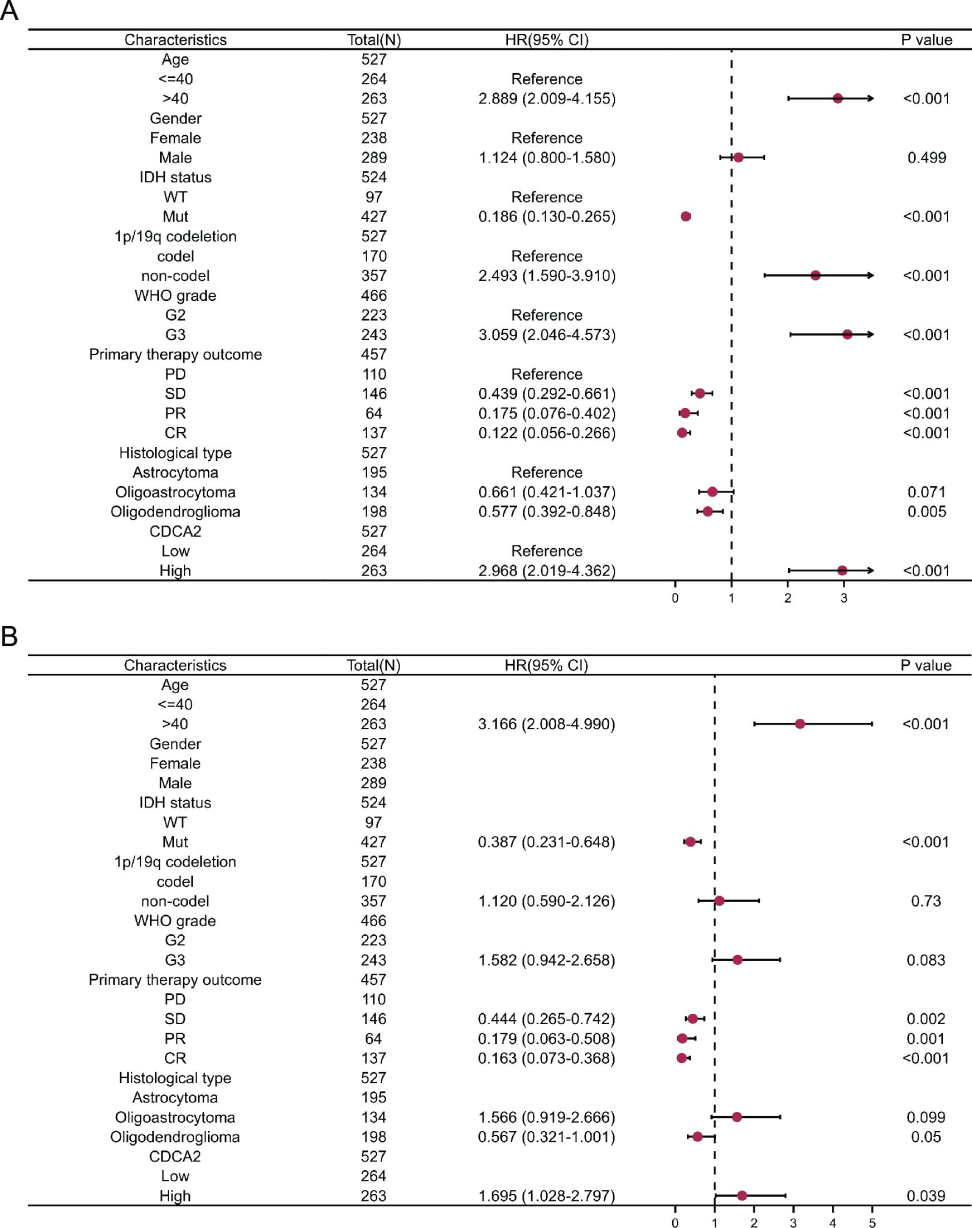
Univariate and multivariate Cox regression analyses of clinical features of LGG associated with OS.

We explored the prognostic variables linked with LGG OS using univariate and multivariate analysis. Age, IDH status, 1p/19q codeletion, WHO grade, primary therapy outcome, histological type, and CDCA2 were identified as independent prognostic factors for LGG in the univariate regression Cox model (Fig 6A). A multivariable analysis showed that age, IDH status, primary therapy outcome, and CDCA2 were independently correlated with LGG OS (Fig 6B).

### Construction and validation of nomogram

**Fig 7:**
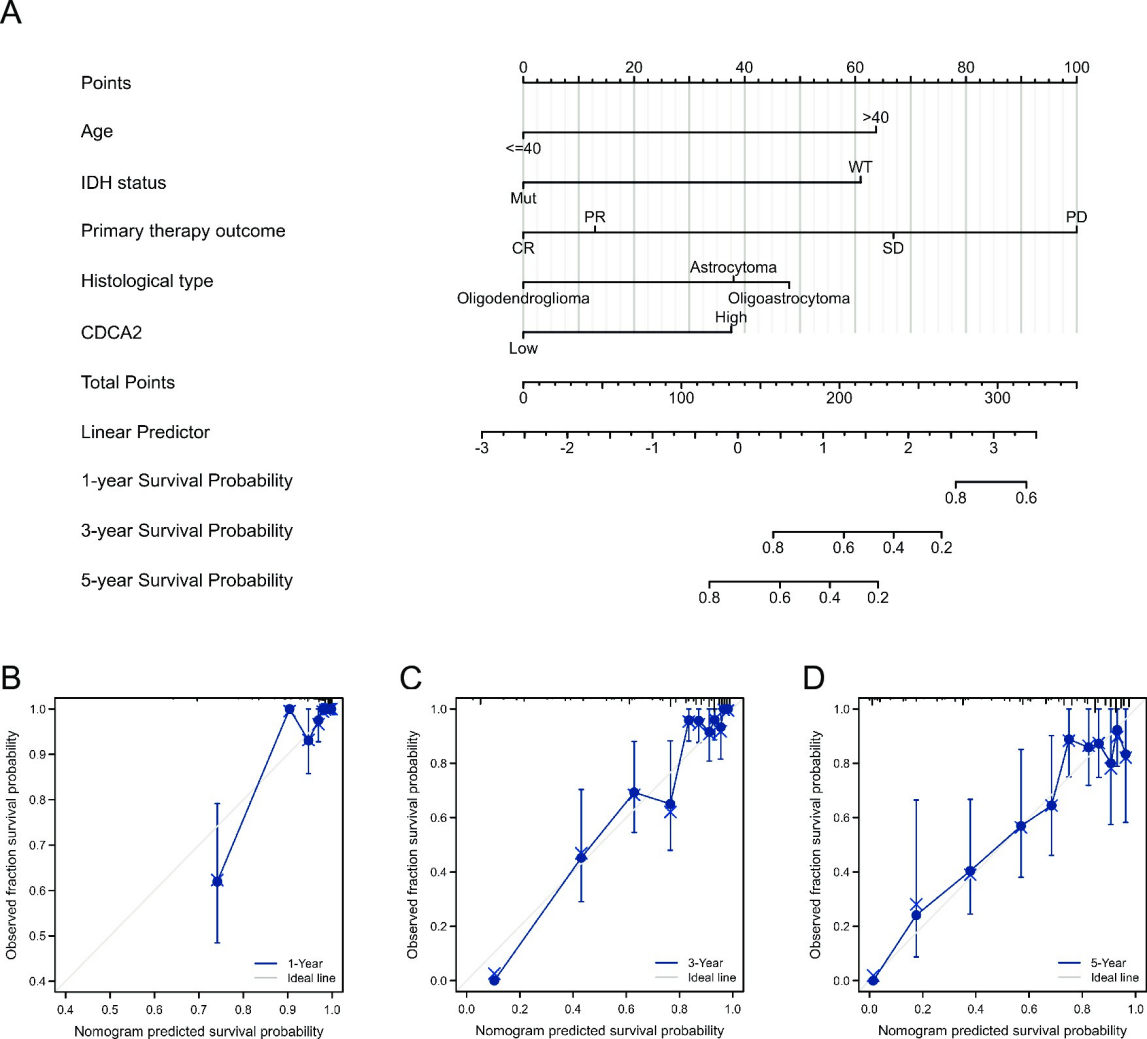
Nomogram model and calibration curves. (A) Nomogram model predicting the 1-, 3- and 5-year OS in patients with LGG. The calibration curves for nomogram at **(**B**)** 1 year, (C) 3 years and (D) 5 years.

Based on the variables that were statistically significant in the multivariate analysis, we created a prognostic nomogram to estimate 1-, 3-, and 5-year survival probabilities in order to produce a therapeutically practical strategy for predicting the prognosis of LGG patients (Fig 7A). Each regression coefficient in multivariate analysis was converted to a 0‐ to 100‐point scale. A nomogram with five variables, assigning a score to each covariate by plotting vertical lines down to the axis markers. Individual probabilities for overall survival at 1, 3, and 5 years can be calculated by adding the entire scores and positioning them on the total score scale. The discrimination and calibration of the nomogram were evaluated using the concordance index (C‐index) and calibration plots. The C-index of the nomogram was 0.850 (95% CI = 0.833–0.867, p< = 2e-16), which indicated satisfactory discriminative ability of the nomogram. The calibration plots showed favorable consistency between the prediction of the nomogram and actual observations. (Fig 7B–7D).

### Enrichment analyses

**Fig 8:**
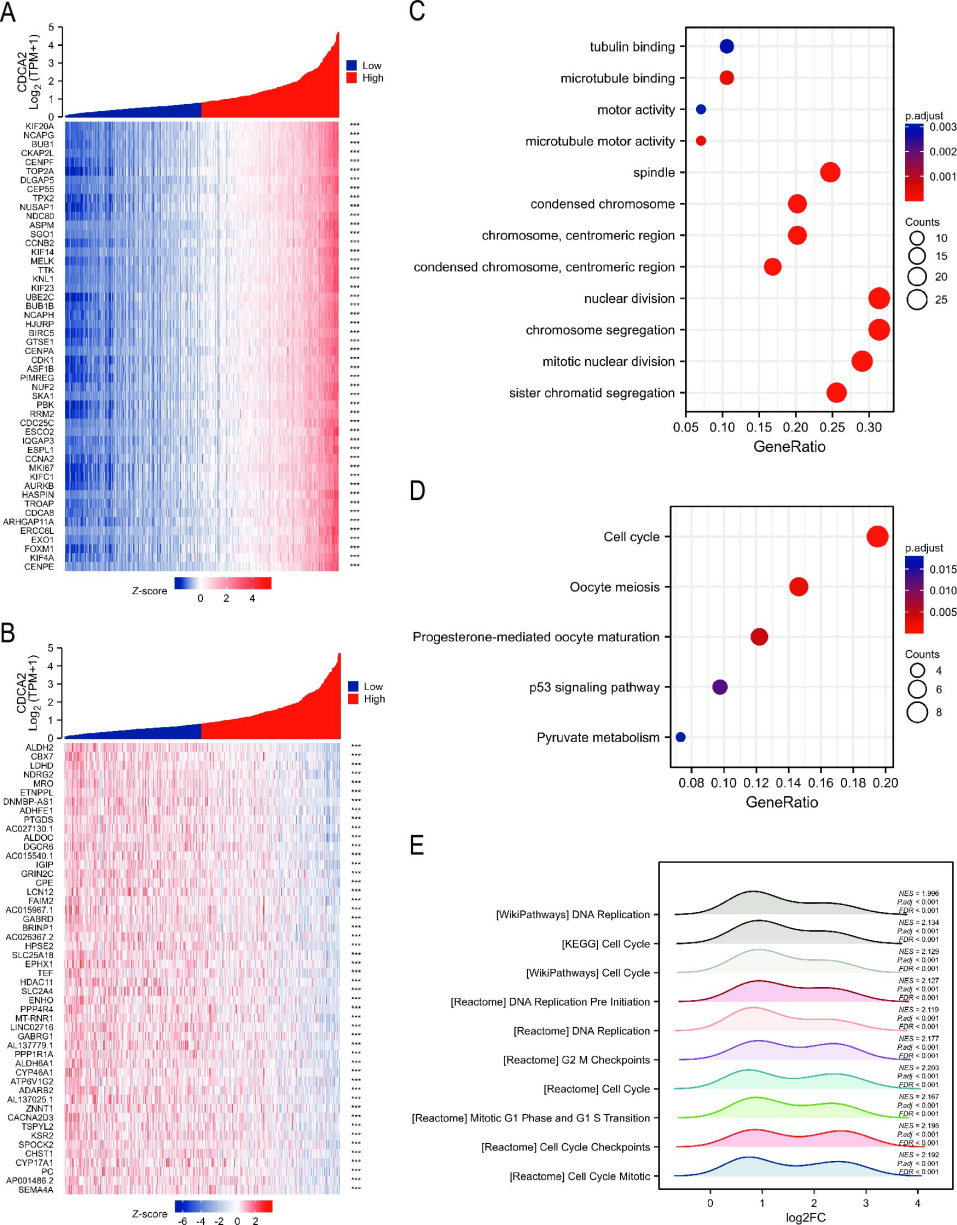
Enrichment analysis of CDCA2 in LGG. (A, B) Top 100 positive and negative co-expressed genes with CDCA2 in LGG. (C, D) GO and KEGG analyses of CDCA2 related genes. (E) GSEA for differentially expressed genes of CDCA2.

We screened for genes that were co-expressed with CDCA2 in LGG. The top 100 positive and negative co-expressed genes were shown in Fig 8A and 8B. Using these 100 co-expressed genes, KEGG and GO analyses were performed. GO enrichment analysis revealed that these genes were mostly enriched in tubulin binding, spindle, condensed chromosome, nuclear division, and chromosomal segregation (Fig 8C). The results of KEGG showed that these genes were involved in cell cycle, oocyte meiosis, progesterone-mediated oocyte maturation, p53 signaling pathway, and pyruvate metabolism (Fig 8D). In addition, GSEA was used to examine the pathways in which CDCA2 may be implicated, and Fig 8E displays the top ten highly enriched pathways. These pathways were mainly enriched in cell cycle, mitotic, and DNA replication.

### Construction of ceRNA network

**Fig 9:**
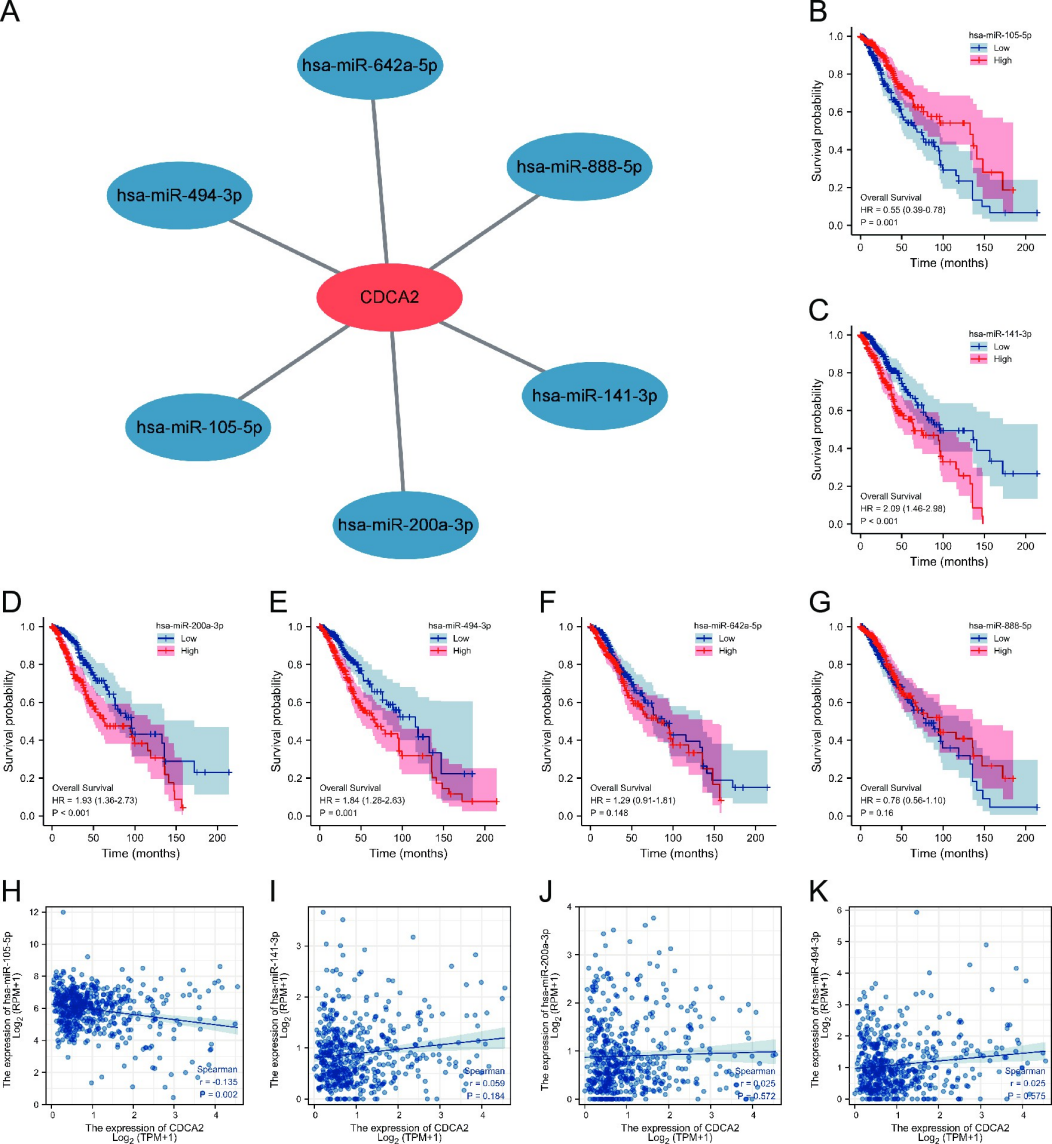
miRNAs binding to CDCA2 in LGG. (A) 6 miRNAs that might bind to CDCA2; (B-G) Survival analyses of 6 miRNAs in LGG; (H-K) Correlation between CDCA2 and miRNAs.

We predicted the miRNAs that might bind to CDCA2 through the starbase database. The result showed that 6 miRNAs might bind to CDCA2, including miRNA-105-5p, miRNA-141-3p, miRNA-200a-3p, miRNA-494-3p, miRNA-642a-5p, and miRNA-888-5p (Fig 9A). The miRNA-seq data was downloaded from TCGA, and survival analyses of these 6 miRNAs were performed. miRNA-105-5p, miRNA-141-3p, miRNA-200a-3p, and miRNA-494-3p were found to be significantly associated with LGG OS (Fig 9B–9G). Correlation analysis revealed that only miRNA-105-5p was negatively correlated with CDCA2 expression (Fig 9H–9K).

**Fig 10:**
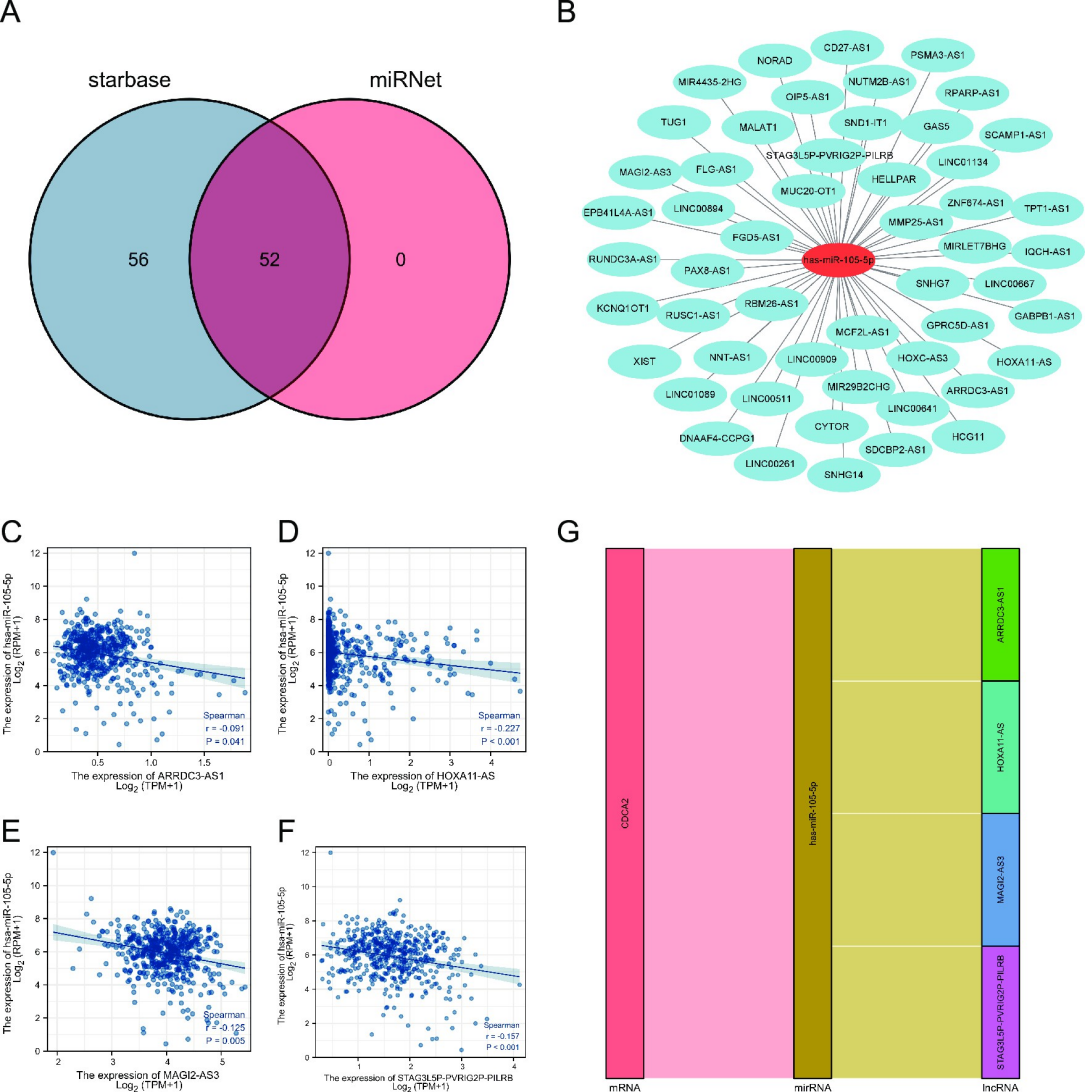
lncRNAs binding to miRNA-105-5p in LGG. (A, B) 52 lncRNAs predicted to interact with miRNA-105-5p; (C-F) Correlation between lncRNAs and miRNA-105-5p; (G) a lncRNA-miRNA-mRNA ceRNA network.

Subsequently, 52 lncRNAs were predicted by the starbase and miRNet databases (Fig 10A and 10B). Survival analysis showed that 22 lncRNAs were remarkably related to OS of LGG (S5 Fig). Among 22 lncRNAs, ARRDC3-AS1, HOXA11-AS, MAGI2-AS3, and STAG3L5P-PVRIG2P-PILRB were negatively correlated with miRNA-105-5p (Fig 10C–10F). So, a lncRNA-miRNA-mRNA ceRNA network was constructed (Fig 10G).

### Association with m6A RNA methylation regulators in LGG

**Fig 11:**
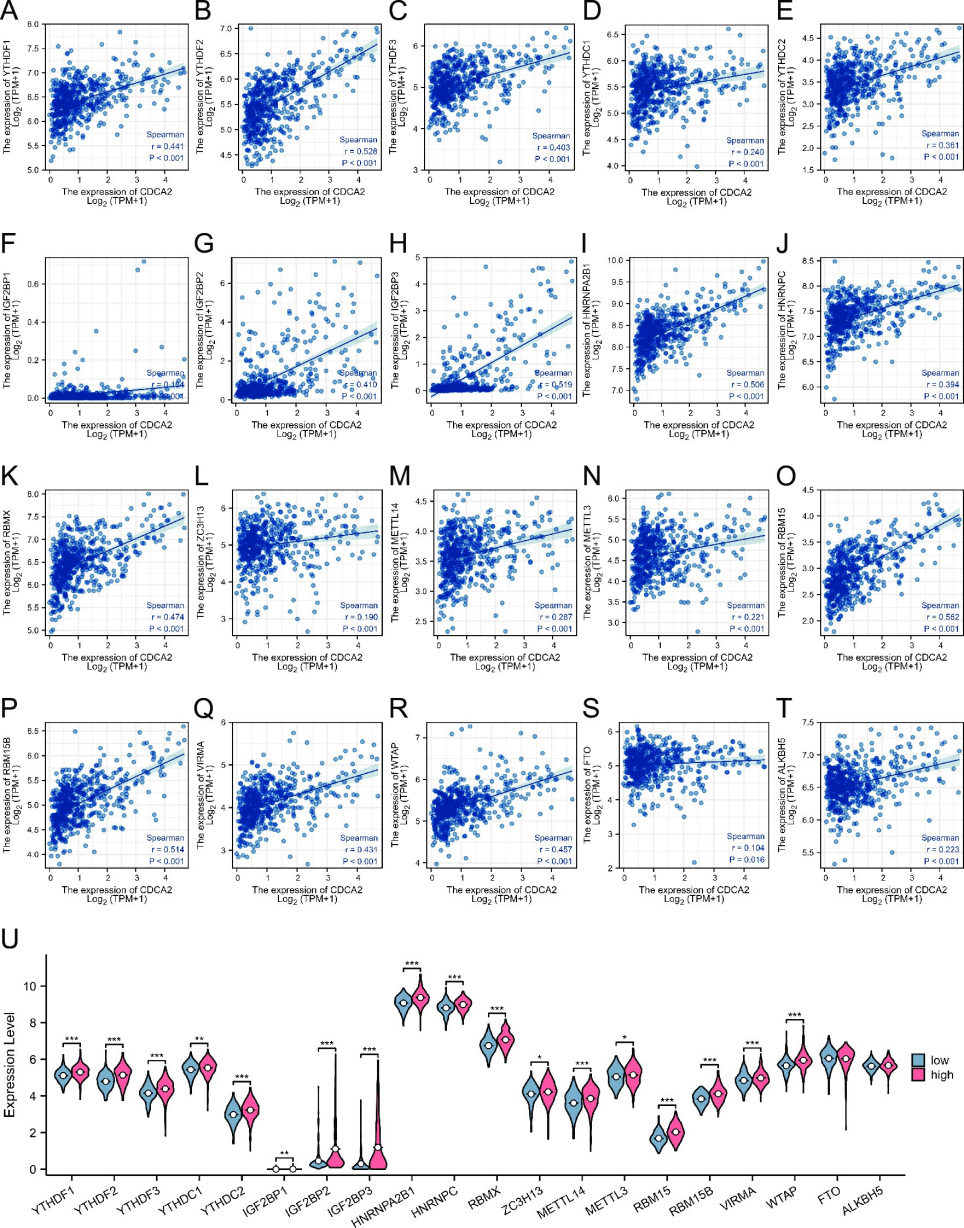
Associations between CDCA2 expression and m6A-related genes in LGG. (A-T) The correlation between CDCA2 and m6A related genes; (U) The differential expression of m6A related genes in the high and low CDCA2 expression groups in LGG (*p < 0.05, **p < 0.01, ***p < 0.001).

Recent studies indicate that m6A epigenetic modification plays an important role in tumor occurrence and development. The relationships between CDCA2 expression and m6A-related genes were explored in LGG. Fig 11A–11T showed that 20 m6A-related genes were significantly positively related to CDCA2 expression in LGG, including YTHDF1 (r = 0.441, p < 0.001),YTHDF2 (r = 0.528, p < 0.001), YTHDF3 (r = 0.403, p < 0.001),YTHDC1 (r = 0.240, p < 0.001), YTHDC2 (r = 0.361, p < 0.001), IGF2BP1 (r = 0.184, p < 0.001), IGF2BP2 (r = 0.410, p < 0.001), IGF2BP3 (r = 0.519, p < 0.001), HNRNPA2B1 (r = 0.506, p < 0.001), HNRNPC (r = 0.394, p < 0.001), RBMX (r = 0.474, p < 0.001), ZC3H13 (r = 0.190, p < 0.001), METTL14 (r = 0.287, p < 0.001), METTL3 (r = 0.221, p < 0.001), RBM15 (r = 0.562, p < 0.001), RBM15B (r = 0.514, p < 0.001), VIRMA (r = 0.431, p < 0.001), WTAP (r = 0.457, p < 0.001), FTO (r = 0.104, p = 0.016), and ALKBH5 (r = 0.223, p < 0.001). Additionally, the differential expression of 20 m6A-related genes in LGG was compared between the CDCA2 high and low expression groups. All of the m6A-related genes except for FTO and ALKBH5 were found to be significantly up-regulated in the CDCA2 high expression group (Fig 11U).

### Relationship with immune infiltration

**Fig 12:**
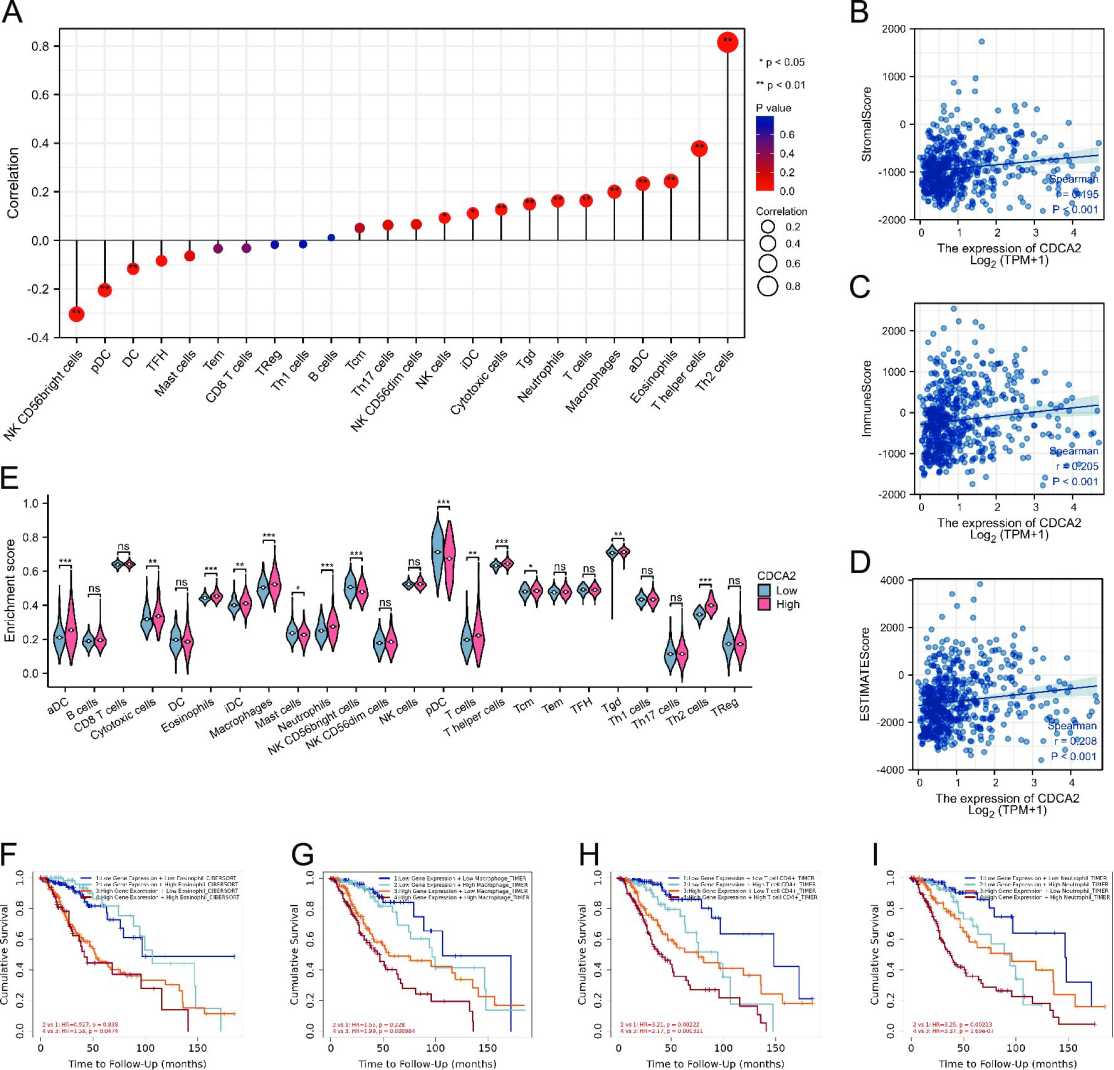
Associations between CDCA2 expression and immune cell infiltration in LGG. (A) The correlation between CDCA2 and immune cell infiltration; (B, C, D) The correlation between CDCA2 and stromalscore, immune score and estimate score; (E) The differential expression of immune cells in the high and low CDCA2 expression groups in LGG; The relationship between the different infiltration levels of immune cells and the prognosis of patients with LGG (Fig 12F–12I) (*p < 0.05, **p < 0.01, ***p < 0.001).

A series of analyses were carried out to determine the relationship between CDCA2 expression and immune infiltration levels in LGG. CDCA2 was positively related to NK cells, iDC, Cytotoxic cells, Tgd, Neutrophils, T cells, Macrophages, aDC, Eosinophils, T helper cells, and Th2 cells and negatively related to DC, pDC, and NK CD56bright cells (Fig 12A). The expression of CDCA2 was found to be significantly positively related to the LGG microenvironment stomal score, immune score, and estimate score (The stromal and immune scores were used to predict the levels of infiltrating stromal and immune cells, which formed the basis for the estimated score to infer tumor purity in tumor tissue [15]) (Fig 12B–12D). Furthermore, the differences in immune cell infiltration between the CDCA2 high and low groups were investigated. T cells, aDC, Cytotoxic cells, Eosinophils, iDC, Macrophages, Neutrophils, T helper cells, Tcm, Tgd, and Th2 cells were more abundant in LGG patients with high CDCA2 levels (Fig 12E). And survival analysis showed that in the CDCA2 high expression group, compared with Eosinophil, Macrophage, Neutrophil and T cell CD4+ low infiltration levels, LGG patients with Eosinophil, Macrophage, Neutrophil and T cell CD4+ high infiltration levels had worse prognoses. (Fig 12F–12I).

## Discussion

In our work, CDCA2 expression in tumors and normal tissues was compared. Fig 1 shows that CDCA2 expression levels were elevated in 28 types of malignancies and reduced in two types of cancer. Our findings imply that CDCA2 may play a role in carcinogenesis and serve as an oncogene in most tumors. CDCA2 expression was also observed to vary substantially across immunological subtypes of 23 different malignancies and molecular subtypes of 12 distinct tumors. Our findings provide light on the relationship between CDCA2 and immunological and molecular subtypes, which might lead to the development of new immunotherapies and target therapeutics.

Next, in order to evaluate the diagnostic value of CDCA2 in tumors, we performed ROC curve analyses. CDCA2 demonstrated excellent predictive performance (AUC > 0.9) in 20 distinct types of cancer, according to the findings of the ROC curve. To get insight into the relationship between CDCA2 and the survival of tumor patients, survival analyses using the KM curves, were performed. In 11 different kinds of cancer, survival study revealed that the amount of CDCA2 expression was shown to be connected with OS, DSS, and PFI. All these results suggest that CDCA2 had an extensive diagnostic and prognostic value in cancers.

Studies have found that CDCA2 is related to the occurrence and development of various cancers. CDCA2 upregulation may directly target CCND1 to stimulate PI3K/AKT pathway to be activated, thereby promoting CRC cell proliferation and carcinogenesis [16]. CDCA2 is overexpressed in prostate cancer patients and regulates cell proliferation. It is activated by hypoxia and is controlled directly by the HIF-1/Smad3 complex [17]. CDCA2 promotes the pathogenesis of HCC by inhibiting the p53-PUMA/NOXA signaling pathway. And overexpression of CDCA2 is related to poor stage, pathological grade, and clinical outcome [18].

CDCA2 has not been reported in the literature in LGG, so we conducted further in-depth analysis in LGG. A stratified analysis of clinical features subsequently revealed its prognostic implications in subgroups with LGG. The high expression of CDCA2 in the subgroups of IDH status (Mut), WHO grade (G3), and histological type (astrocytoma and oligodendroglioma) decreased overall survival of LGG patients, allowing researchers to more accurately determine the prognosis of LGG patients and conduct targeted therapy studies for subgroups. In addition, we constructed a nomogram to assess the prognosis of LGG patients. This nomogram exhibits good predictive power and will allow doctors and patients to make more personalized prognostic judgments.

To explore mechanism of CDCA2, GO, KEGG pathway analysis, and GSEA were performed. Our findings support previous reports that CDCA2 is primarily involved in cell cycle, mitosis, chromatin condensation, DNA replication, and p53 pathway, etc [2, 19]. CDCA2 plays an important role in cell cycle progression. It is reported that the level of CDCA2 is a key determinant in the DNA damage checkpoint’s activation [2]. Two master kinases known as Ataxia telangietisa mutant (ATM) and ataxia-telangiectasia mutated related (ATR) are responsible for regulating checkpoint activation [20]. Disruptions in the structure of the chromatin and DNA double-strand breaks, activate the ATM protein, which in turn phosphorylates p53 at Ser 15 and inhibits MDM2 from attaching to p53 [21]. DNA damage-induced cell cycle checkpoints transiently delay cell cycle progression in proliferating cells, which may induce cell cycle arrest at specific phases [22]. Vagnarelli et al. also reported that CDCA2 acts as a key regulator in chromatin remodeling by targeting PP1 for the de-phosphorylation of histone H3 [3]. CDCA2’s significance in cancer development has recently received increasing attention [8, 9].

In order to explore the upstream regulatory mechanism, we constructed a ceRNA network. One miRNA (miRNA-105-5p) and four lncRNAs (ARRDC3-AS1, HOXA11-AS, MAGI2-AS3, and STAG3L5P-PVRIG2P-PILRB) were identified. Serum extracellular vesicle-derived miRNA-105-5p can be transferred to ESCC cells and contribute to ESCC proliferation through targeting SPARCL1 and regulating the FAK/Akt signaling pathway. It has been reported that HOXA11-AS could play an important role in many types of tumors. For example, HOXA11-AS can serve as an oncogene in glioma, breast cancer, and non-small cell lung cancer [23–25]. MAGI2-AS3 is found to be less expressed in glioma tissues [26]. ARRDC3-AS1 and STAG3L5P-PVRIG2P-PILRB ARRDC3-AS have not been reported in the literature.

N6-methyladenosine (m6A) RNA methylation, first discovered in the 1970s, is the most prevalent dynamic and reversible epigenetic modification in mRNAs [27]. m6A modification abnormality in RNA is strongly linked to the initiation and progression of a wide range of cancers. In various malignancies, m6A-related genes (writers, erasers, and readers) have been shown to either accelerate or repress carcinogenesis [28–31]. Upregulation or downregulation of certain m6A-related genes and activation or inhibition of certain m6A regulators can enhance the sensitivity of tumors to treatment [32]. It’s becoming more and more clear that methylation regulators of the area of m6A in RNA, such as FTO and YTHDF2, may have an impact on glioma cell proliferation, carcinogenesis, proliferation and growth and invasion by influencing the levels of mRNA expression in their target genes. According to this research, an important glioma-specific therapeutic target and clinical prognostic indicator has been identified as the m6A-target gene axis [33–37].

There is no report on the association between CDCA2 and m6A. According to the findings of our research, the expression of CDCA2 in LGG was shown to be substantially correlated with 20 m6A-related genes. We have reason to assume that m6A affects the translation and stability of CDCA2, which in turn contributes to the onset and progression of LGG. This also provides us with a fresh path to examine the mechanism of action of CDCA2, which is quite helpful.

Immune cell infiltration is a characteristic of the majority of solid tumors. Tumor growth, progression, and metastasis are all influenced by the interplay between immune cells and tumor cells in the tumor microenvironment [38]. It’s true that immunotherapy has helped a lot of people with cancer in the last several years. As a promising new therapy option for cancer, immune checkpoint inhibitors have recently gained attention [39]. Nevertheless, immunosuppressive tumor microenvironment induced by substantial infiltration of immune cells usually leads to immunotherapy resistance [40]. As a result, a significant number of studies have concentrated their efforts on finding new targets linked to high levels of immune cell infiltration in gliomas. For instance, increased numbers of costimulatory checkpoints SLAMF8 was implicated in the aggravation of immunosuppression [41]. Our study found that CDCA2 high expression group of LGG had a considerably higher infiltration level of 11 kinds of immune cells, and the expression level of CDCA2 was significantly positively connected with the stomal score, immunological score, and estimated score. In the CDCA2 high expression group, the high infiltration levels of Eosinophil, Macrophage, Neutrophil and T cell CD4+ were associated with poor prognoses of LGG patients. Our analysis indicated that elevated CDCA2 expression could promote immune infiltration in LGG. As a result, we would like to conduct further research on the impact of CDCA2 inhibition on immune cell infiltration and survival time in LGG patients.

Our findings show that CDCA2 might be a diagnostic and prognostic biomarker and therapeutic target in pan-cancer, particularly in LGG. Furthermore, we investigate the CDCA2 mechanism in LGG. We construct a ceRNA network and uncover its upstream and downstream regulatory relationships. Our research also showed that CDCA2 is associated with m6A modification and immune infiltration in LGG. This gives a bioinformatic foundation for further study. The conclusions of this study were based on bioinformatics analysis, and as a result, they need to be further confirmed.

## Supporting information

S1 Fig The ROC curve analysis of CDCA2 in pan-cancer (0.9>AUC>0.7). (A) ACC; (B) DLBC; (C) HNSC; (D) KIRP; (E) PRAD; (F) SKCM; (G) TGCT; (H) THCA; (I) THYM.
(TIF)

S2 Fig Disease specific survival curves of CDCA2 in cancers. (A) ACC; (B) BLCA; (C) ESCC; (D) KIRC; (E) KIRP; (F) LGG; (G) LIHC; (H) LUAD; (I) MESO; (J) PAAD; (K) SARC.
(TIF)

S3 Fig Progress free interval curves of CDCA2 in cancers. (A) ACC; (B) BLCA; (C) ESCC; (D) KIRC; (E) KIRP; (F) LGG; (G) LIHC; (H) LUAD; (I) MESO; (J) PAAD; (K) SARC.
(TIF)

S4 Fig OS analysis of CDCA2 in clinical subgroups of LGG. (A) 1p/19q codeletion (codel); (B) 1p/19q codeletion (non-codel); (C) IDH status (Mut); (D) WHO grade (G3); (E) age > 40; (F) age ≤ 40; (G) Gender (Female); (H) Gender (Male); (I) Histological type (Astrocytoma); (J) Histological type (Oligodendroglioma); (K) Primary therapy outcome (PD); (L) Primary therapy outcome (SD).
(TIF)

S5 Fig Overall survival curves of lncRNAs in LGG. (A-V) 22 lncRNAs were significantly associated with OS of LGG.
(TIF)
