# Supplementary figures and images for: A pan-cancer analysis reveals the diagnostic and prognostic role of CDCA2 in low-grade glioma
Source: PLoS One. 2023 Sep 21;18(9):e0291024. doi: 10.1371/journal.pone.0291024 (PMC10513342; doi:10.1371/journal.pone.0291024)

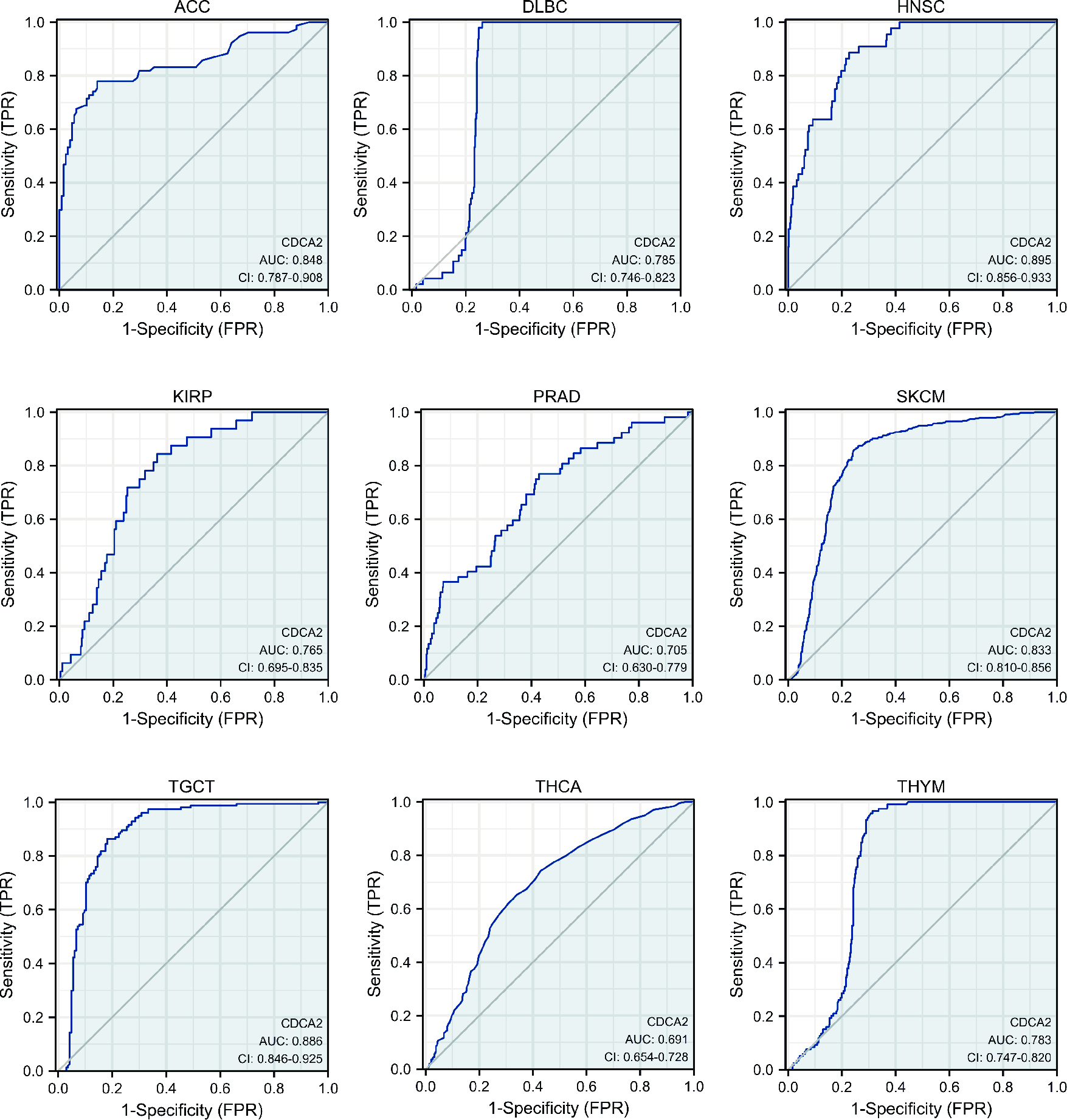

Supplement: S1 Fig — (A) ACC; (B) DLBC; (C) HNSC; (D) KIRP; (E) PRAD; (F) SKCM; (G) TGCT; (H) THCA; (I) THYM. (TIF) [file pone.0291024.s001.tif]

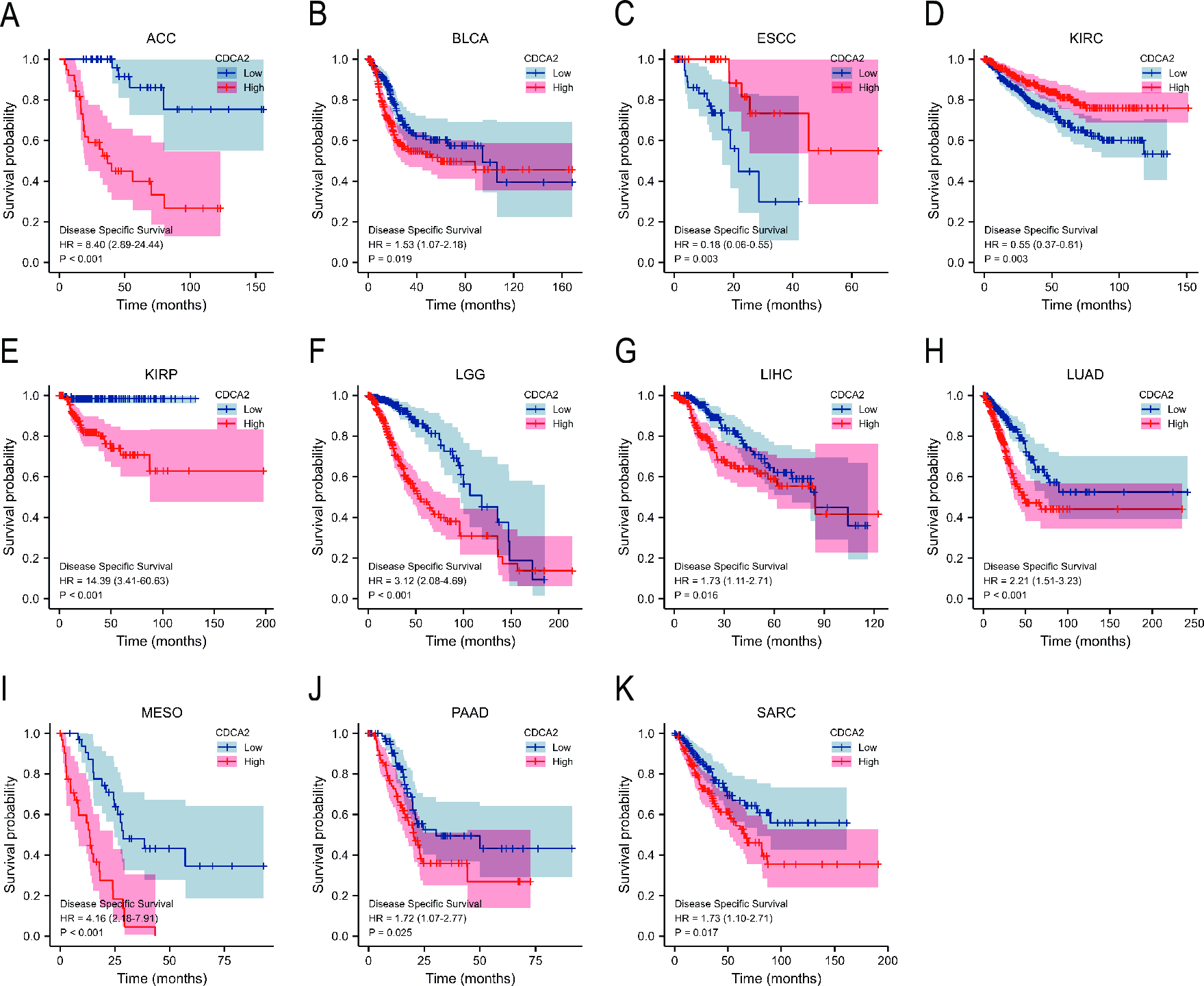

Supplement: S2 Fig — (A) ACC; (B) BLCA; (C) ESCC; (D) KIRC; (E) KIRP; (F) LGG; (G) LIHC; (H) LUAD; (I) MESO; (J) PAAD; (K) SARC. (TIF) [file pone.0291024.s002.tif]

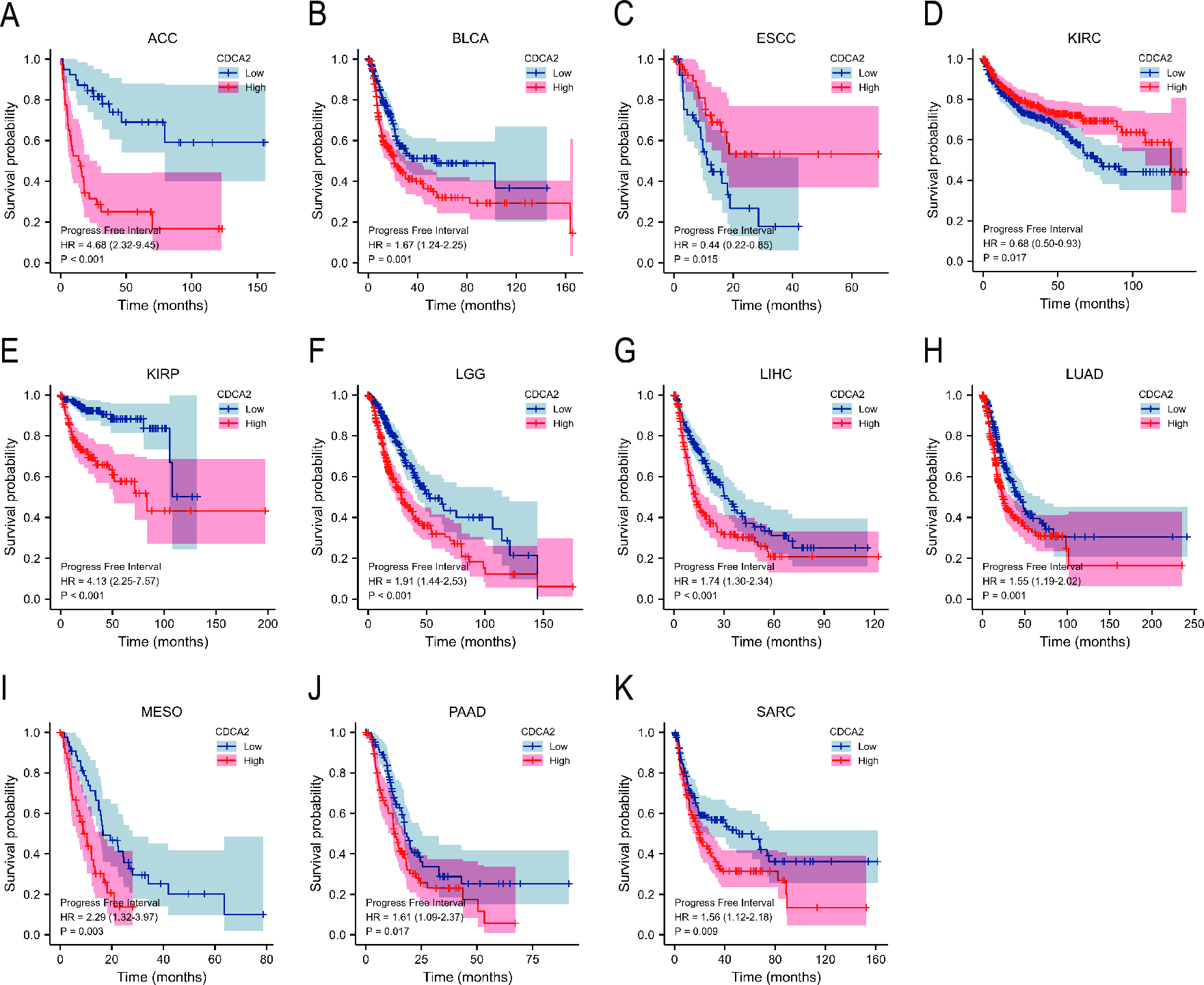

Supplement: S3 Fig — (A) ACC; (B) BLCA; (C) ESCC; (D) KIRC; (E) KIRP; (F) LGG; (G) LIHC; (H) LUAD; (I) MESO; (J) PAAD; (K) SARC. (TIF) [file pone.0291024.s003.tif]

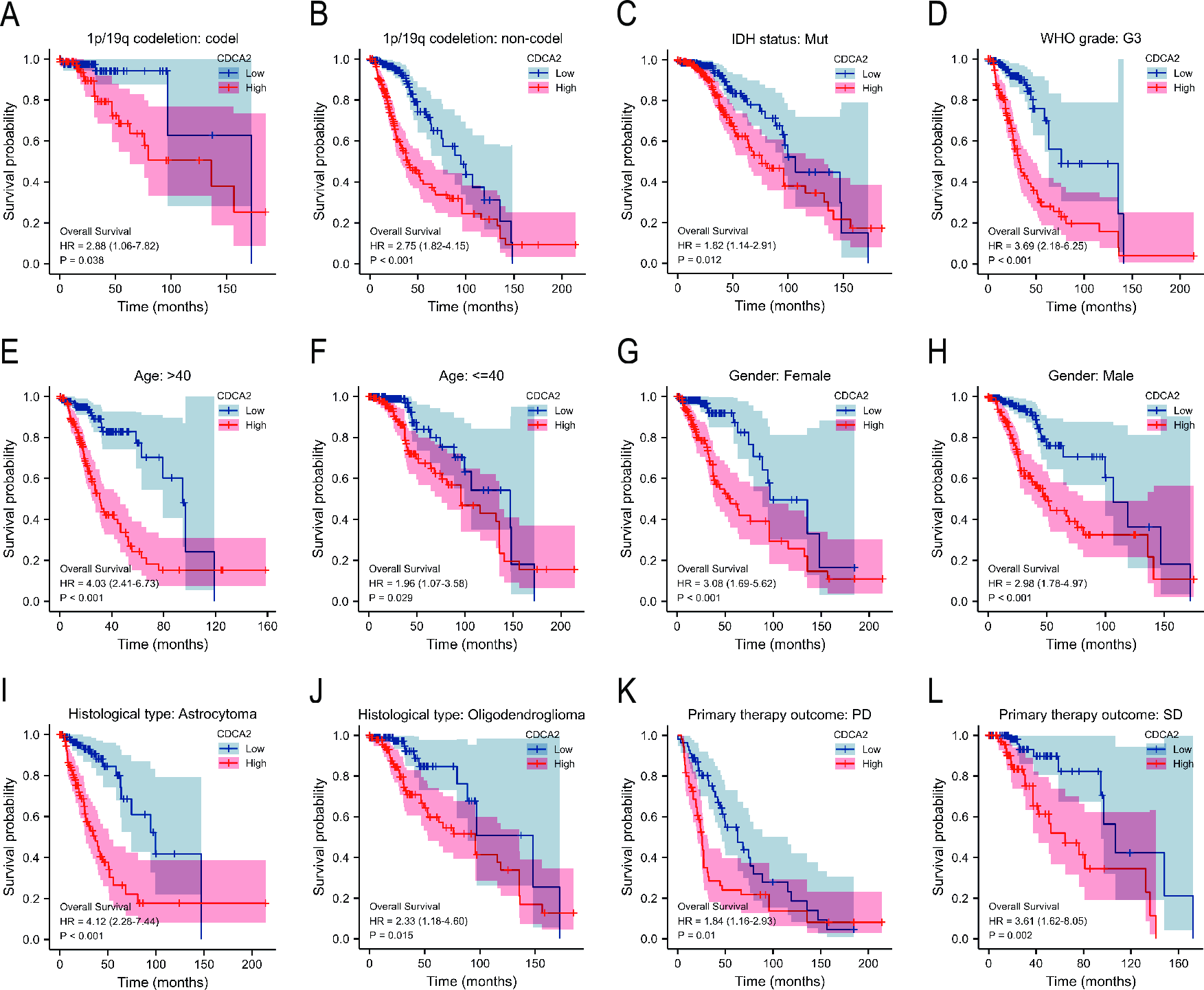

Supplement: S4 Fig — (A) 1p/19q codeletion (codel); (B) 1p/19q codeletion (non-codel); (C) IDH status (Mut); (D) WHO grade (G3); (E) age > 40; (F) age ≤ 40; (G) Gender (Female); (H) Gender (Male); (I) Histological type (Astrocytoma); (J) Histological type (Oligodendroglioma); (K) Primary therapy outcome (PD); (L) Primary therapy outcome (SD). (TIF) [file pone.0291024.s004.tif]

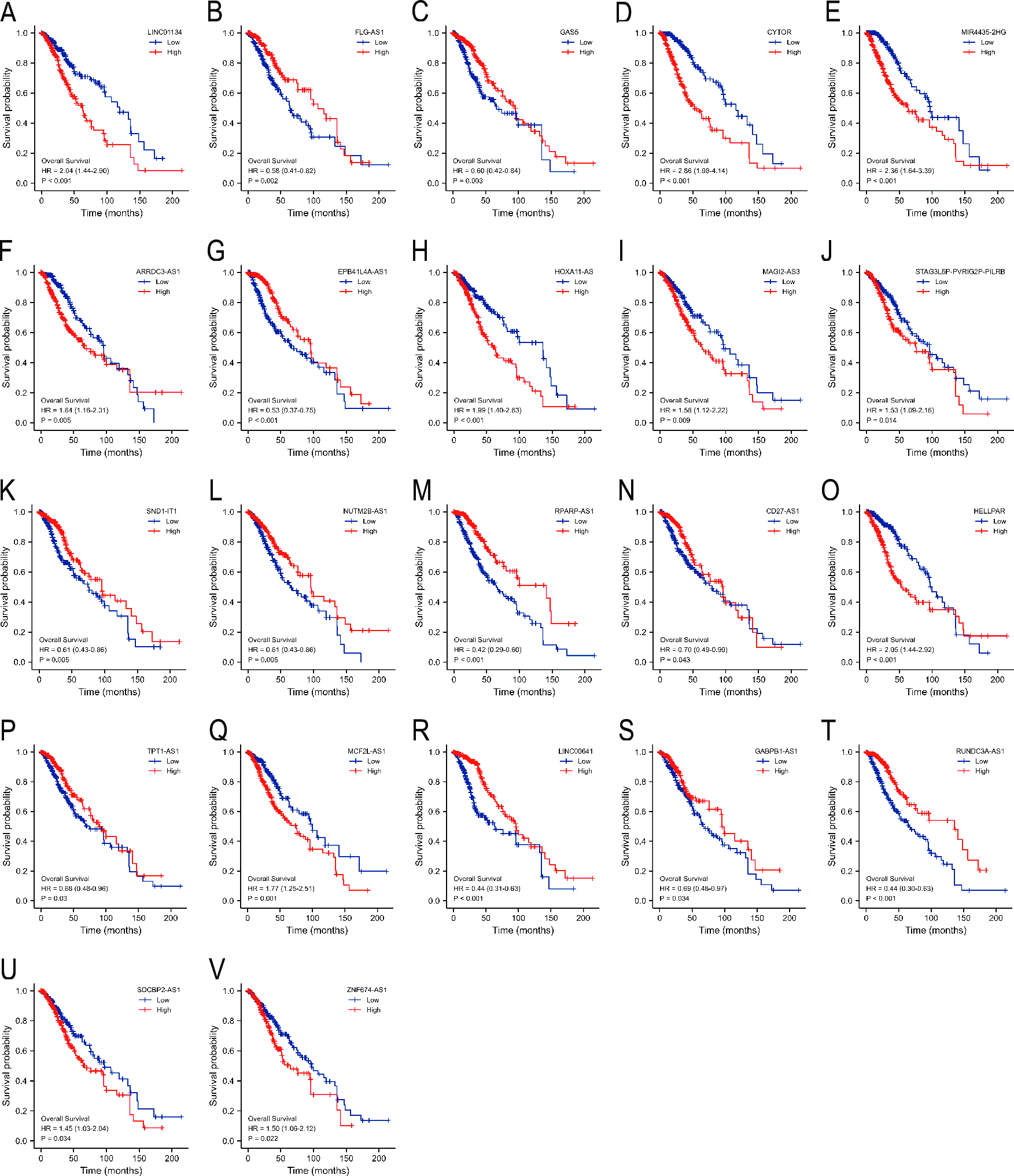

Supplement: S5 Fig — (A-V) 22 lncRNAs were significantly associated with OS of LGG. (TIF) [file pone.0291024.s005.tif]
